# Detecting the Physicochemical Transformations in Solid Drug Products Stored for Long Periods of Time—Insights into DSC Application

**DOI:** 10.3390/molecules31081280

**Published:** 2026-04-14

**Authors:** Edyta Leyk, Tomasz Konarski, Marek Wesolowski

**Affiliations:** Department of Analytical Chemistry, Medical University of Gdansk, Gen. J. Hallera 107, 80-416 Gdansk, Poland; edyta.leyk@gumed.edu.pl (E.L.); t.konarski24@gmail.com (T.K.)

**Keywords:** solid drug products, long term storage, drug products degradation, physicochemical transformations, differential scanning calorimetry, principal component analysis

## Abstract

Since differential scanning calorimetry (DSC) is an excellent method for studying phase transformations in the solid state, the purpose of this study was to assess the suitability of DSC as a method for detecting physicochemical transformations occurring in solid drug products during storage, extending also beyond their expiration date. Based on the DSC measurements of 34 commercial drug products, they were divided into three groups characterized by the fact that the DSC curves show: (I) as dominant endothermic peaks reflecting active pharmaceutical ingredients (APIs) melting, with no additional peaks from excipients, (II) in addition to the peaks reflecting APIs melting, additional peaks related to the excipients, and (III) two peaks characteristic of lactose monohydrate dehydration and melting. Analysis of the temperature ranges and the shape of the DSC peaks showed no significant differences between the six series of measurements performed between 2011 and 2022, suggesting that physicochemical changes in drug products could not be detected during storage. Only the use of principal component analysis (PCA) made it possible to separate the DSC curves obtained during long-term storage of drug products. This allows DSC to be used to detect the first signs of deterioration, but only for drug formulations in group one. Of the drug products in groups two and three, this is only possible for 14 products. It follows that the suitability of DSC for identifying physicochemical changes in products stored for long periods of time is affected by the API content and complex composition of the tablet matrix.

## 1. Introduction

From a public health perspective, the quality, safety, and efficacy of drugs used in the treatment, prevention, or diagnosis of diseases are key issues [1,2]. One of the factors influencing these requirements for drugs entering the pharmaceutical market is the stability of drug substances (active pharmaceutical ingredients, APIs). It should be noted that a large group of APIs are characterized by low stability [3,4]. Overall, the stability of API is significantly affected by atmospheric factors (moisture, oxygen, light, temperature). For this reason, it is necessary to select appropriate excipients and dosage forms through which APIs contained in drug products (drug formulations, pharmaceutical products) achieve resistance to external factors.

When considering API stability, it is important to take into account chemical stability, which includes possible changes in the chemical structure of the molecule, as well as physical stability, which is related to changes in the physical properties of the substance [3,4]. The consequences of these unfavorable changes may include a decrease in the content or polymorphic transformation of the API, and thus a decrease in the therapeutic value of the drug product. Therefore, the stability determines the permissible expiration date of a drug, i.e., the time during which the content of the API will not decrease below a certain value (usually below 95%), the drug product will meet all the quality requirements for the drug formulation, and the degradation products will not be toxic [5]. The risk of a potential decrease in the API content and therapeutic value of the drug product and the appearance of toxic API degradation products make it mandatory to control the consistency of the drug product with the regulations that have been established for it. This refers to both distribution and storage of drug products.

The acceptable expiration date of most solid drug products is three years, after which time they should be disposed of. To determine expiration date, high-performance liquid chromatography (HPLC) is most often used, which makes it possible, among other things, to assess API content and identify API degradation products [6,7]. However, HPLC requires carrying out solid dosage forms into solution, which eliminates the possibility of obtaining data on the forms in which APIs are present in the drug products studied, i.e., amorphous or crystalline form; polymorphic modifications; anhydrous form; hydrate; or solvate. Since these are key data that significantly affect the stability and bioavailability of solid drug formulations, potential changes should be monitored, eliminating drug products that do not meet the manufacturer’s requirements. A useful method for this study is differential scanning calorimetry (DSC), an excellent tool for studying phase transformations in the solid state [8]. Therefore, the purpose of this study was to determine the usefulness of DSC as a tool for detecting whether and what physical transformations and chemical processes occur in drug products during storage, extending also beyond their expiration date. Hence, solid dosage forms selected for the study were stored in a drug warehouse under the specified conditions throughout the study period (11 years).

The great utility of DSC in drug testing has been repeatedly confirmed [9,10,11,12]. A key advantage of DSC is its ability to determine the melting point, which is a characteristic value that confirms the identity and quality of an API. DSC is widely used to determine the purity, polymorphic transformations and hydration/dehydration of APIs; incompatibility between ingredients; and as a valuable tool for studying the effects of different experimental conditions on API properties during technological processes in the pharmaceutical industry. However, there are few examples in the literature of the use of DSC in the study of commercial drug products [13], and there is a complete dearth of information on the usefulness of DSC in identifying potential changes resulting from the gradual deterioration of drug products during long-term storage in a drug warehouse. Obtaining such knowledge may provide a starting point for work on the use of DSC as a method for confirming the quality of drug products available on the pharmaceutical market before/after the expiration date. Furthermore, the advantages of using DSC include the fact that samples do not require special preparation for analysis (extraction, separation), a small sample is used, and the analysis time is short, resulting in high measurement sensitivity. A simple DSC measurement procedure allows data on phase transformations and thermal degradation of the studied samples to be obtained without the need to transfer them to a solution (unlike chromatographic and spectrophotometric methods). These clear advantages of DSC justify the need to determine the usefulness of this method for detecting physicochemical transformations that occur in drug products during storage, also extending beyond their expiration date.

The material for the study was commercial pharmaceutical tablets, which constitute so-called solid dosage forms according to pharmaceutical terminology. In addition to APIs, all drug products include excipients [5]. APIs are responsible for the therapeutic activity of tablets, while excipients enable the manufacture of tablets with optimal dosage, bioavailability and expiration date. Aiming to achieve the best possible therapeutic effect, in addition to classic tablets (coated or uncoated), prolonged (controlled) release tablets, capsules containing prolonged-release powders or granules, pellets, dragees or suppositories are also available on the pharmaceutical market. For this reason, in order to obtain the maximum useful information from DSC analyses, commercially available drug products were selected for the study, which differ in terms of dosage forms (coated and uncoated tablets, prolonged-release tablets), type and content of excipients (e.g., fillers, disintegrants, binders, and coatings, in a total amount between 8 and 96% by tablet mass) and containing APIs from different therapeutic groups and in very different concentrations (e.g., hypotensive, anti-inflammatory, and anti-microbial drugs, in an amount between 4.2 and 91.7% by tablet mass). Such a diverse group of drug products undergoing screening should provide reliable data on the usefulness of DSC as a tool for detecting whether and what physical transformations and chemical processes occur in drug products during long-term storage (11 years).

## 2. Results and Discussion

All the drug products studied are tablets (Table 1), of which 21 are classic uncoated tablets, 11 are coated tablets, and two products are prolonged-release tablets. The endo- and exothermic peaks in the DSC curves of these tablets reflect the physical transformations and chemical processes that the tablet ingredients underwent during heating. Therefore, information on the type and content of all ingredients in the tablets, i.e., APIs and excipients, is needed to correctly interpret the DSC data.

Analyzing the information from the leaflets accompanying the drug products studied, it can be concluded that classic uncoated tablets were characterized by a relatively simple formulation. In addition to API, these tablets contained three to six excipients, most often lactose monohydrate, starch (potato, corn, or rice), gelatin, polyvinylpyrrolidone, talc, and magnesium stearate. The coated tablets were characterized by a more complex chemical composition. They differed from the previous group of tablets in that their core, which usually consisted of 3–5 excipients, was covered by a coating composed of several ingredients. In addition to starch, polyvinylpyrrolidone, talc, and magnesium stearate, coated tablets usually contained microcrystalline cellulose, hydroxypropylmethylcellulose, polyethylene glycol, and carboxymethyl starch. In contrast, prolonged-release tablets contained up to 10 excipients, mainly ammonium polymethacrylate, polyvinylpyrrolidone, hydroxypropylmethylcellulose, talc, and magnesium stearate.

### 2.1. DSC of Commercial Drug Products

**Figure 1:**
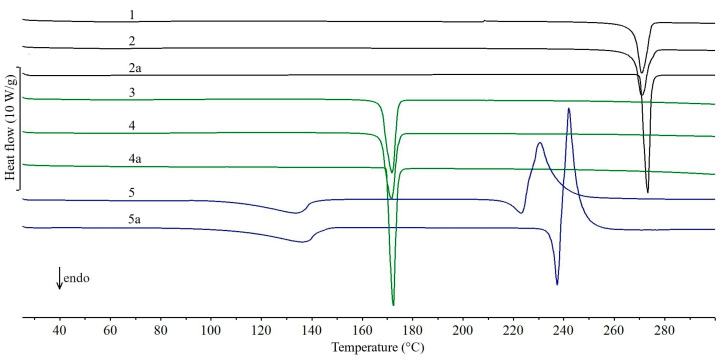
DSC curves of tablets obtained during the first series of experiments (February 2011): Theospirex retard 150 (1), Theospirex retard 300 (2), Paracetamol Biofarm (3), Paracetamol Aflofarm (4), and Pyralgina (5). DSC curves of active pharmaceutical ingredients: theophylline (2a), paracetamol (4a), and metamizole sodium (5a).

**Figure 2:**
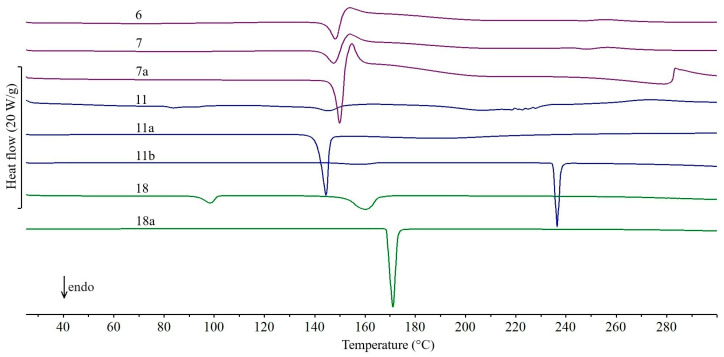
DSC curves of tablets obtained during the first series of experiments (February 2011): Ranigast Polpharma (6), Ranigast Max (7), Coffepirine (11), and Paracetamol Polfa-Łódź (18). DSC curves of active pharmaceutical ingredients: ranitidine hydrochloride (7a), acetylsalicylic acid (11a), caffeine (11b), and paracetamol (18a).

**Figure 3:**
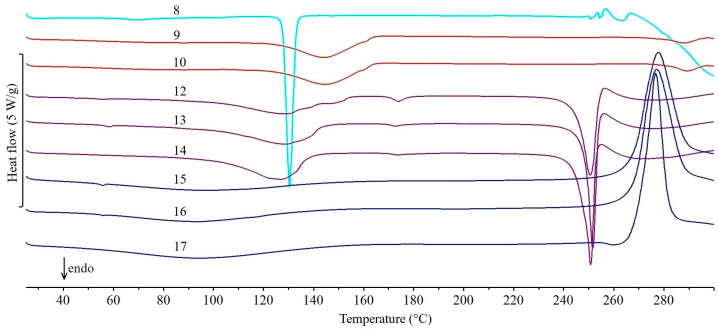
DSC curves of tablets obtained during the first series of experiments (February 2011): Cyclonamine (8), Cipronex 250 (9), Cipronex 500 (10), Heviran 200 (12), Heviran 400 (13), Heviran 800 (14), Nifuroksazyd Hasco (15), Nifuroksazyd 200 Hasco (16), and Nifuroksazyd Richter (17).

**Figure 4:**
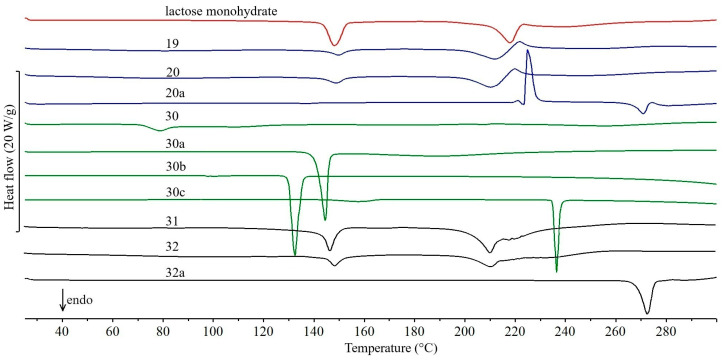
DSC curves of lactose monohydrate and tablets obtained during the first series of experiments (February 2011): Furosemidum Polpharma (19), Furosemidum Polfarmex (20), Etopiryna (30), Tialorid (31), and Tialorid mite (32). DSC curves of active pharmaceutical ingredients: furosemide (20a), acetylsalicylic acid (30a), ethenzamide (30b), caffeine (30c), and hydrochlorothiazide (32a).

**Figure 5:**
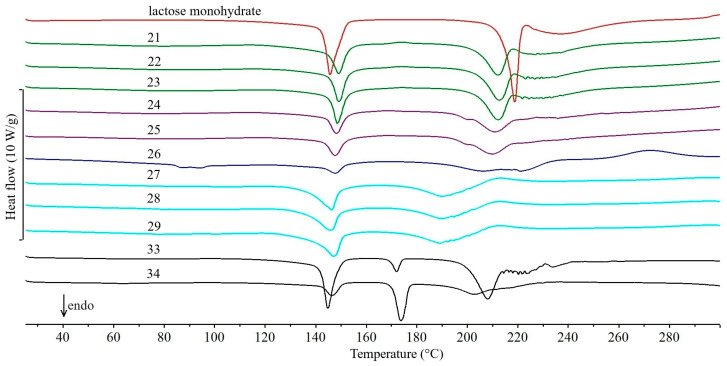
DSC curves of lactose monohydrate and tablets obtained during the first series of experiments (February 2011): Encorton 5 mg (21), Encorton 10 mg (22), Encorton 20 mg (23), Spironol 25 (24), Spironol 100 (25), Metoclopramidum (26), Enarenal 5 (27), Enarenal 10 (28), Enarenal 20 (29), Luminalum Unia 15 (33), and Luminalum Unia 100 (34).

The DSC curves of all the studied drug products obtained during the first series of experiments (February 2011), the active pharmaceutical ingredients (APIs) and lactose monohydrate (excipient) contained in them are shown in Figure 1, Figure 2, Figure 3, Figure 4 and Figure 5. In addition, the parameters of all thermal events for each studied drug product are summarized in Table S1. Interpretation of the curves showed that the tablets studied can be divided into three groups, depending on the DSC data obtained. Accordingly, the following groups of drug products are characterized by the fact that the DSC curves show, in particular: (I) as dominant endothermic peaks reflecting the melting of APIs, without additional peaks from excipients, (II) in addition to the peaks characteristic of the melting of APIs, additional peaks related to the presence of excipients, and (III) two endothermic peaks characteristic of the dehydration and melting of lactose monohydrate.

The first group includes only five drug products in which the API content exceeds 80% of the tablet mass (Table 1). In the DSC curves of tablets containing theophylline—Theospirex retard 150 (Figure 1, curve 1) and Theospirex retard 300 (Figure 1, curve 2)—and two drug products with paracetamol—Paracetamol Biofarm (Figure 1, curve 3) and Paracetamol Aflofarm (Figure 1, curve 4)—only a large and sharply ended endothermic peak due to the melting of the APIs is visible, i.e., theophylline at ~272 °C and paracetamol at ~171 °C (Figure 1, curves 2a and 4a, respectively). The melting temperatures of both APIs are consistent with literature data for the crystalline form I [14,15]. Also, the DSC curve of Pyralgina tablets (Figure 1, curve 5) is consistent with the shape of the DSC curve of metamizole sodium (Figure 1, curve 5a) [16]. This is confirmed by the broadened endothermic peak of API dehydration in the temperature range ~100–140 °C, the sharp endothermic peak due to metamizole sodium melting at 216 °C, and the immediately following strong exothermic effect reflecting API degradation.

In the case of the 13 drug products in group two, in addition to the peaks characteristic of APIs melting, the DSC curves also show additional peaks reflecting the presence of excipients. The DSC curves of the drug products Ranigast Polpharma (Figure 2, curve 6) and Ranigast Max (Figure 2, curve 7) containing ranitidine hydrochloride (Figure 2, curve 7a) primarily reflect the endothermic peak due to melting of the API above 140 °C and the immediately following exothermic peak due to degradation of the API [17]. The small endothermic and exothermic peaks occurring above 240 °C are related to degradation of excipients. The situation is similar for the DSC curves of Cyclonamine tablets (Figure 3, curve 8). In addition to a large and sharp peak at ~130 °C due to melting of etamsylate [18], a small peak at ~70 °C and irregular peaks above 250 °C are visible. These are also the result of degradation of excipients.

In the DSC curves of the drug products Cipronex 250 (Figure 3, curve 9) and Cipronex 500 (Figure 3, curve 10), there is a very weak endothermic peak due to the melting of ciprofloxacin hydrochloride above 278 °C. Its melting point is slightly lower than the literature value of ~303 °C [19]. The DSC peak is small (Δ*H_f_*~10 J/g) despite the fact that the API content of the tablets exceeds 60% (Table 1). A broad and intense peak above 120 °C, unrelated to ciprofloxacin degradation, is also evident. In contrast, the DSC curve of Coffepirine tablets (Figure 2, curve 11) containing acetylsalicylic acid and caffeine (Figure 2, curves 11a and 11b, respectively) shows an endothermic peak above 115 °C. The shape of the peak resembles two overlapping endothermic effects. It reflects the melting of the eutectic mixture formed by the two ingredients and the melting of that part of acetylsalicylic acid that is not involved in the formation of the eutectic. According to literature data, the eutectic formed by acetylsalicylic acid and caffeine melts at 107 °C [20]. It should be noted that the DSC curve of Coffepirine tablets showed no effect at 235 °C, which is associated with caffeine melting [33].

In the drug products Heviran 200, 400, and 800 (Figure 3, curves 12, 13, and 14, respectively), a large and sharp DSC peak due to the melting of acyclovir above 250 °C is followed by a small exothermic peak associated with API degradation [21,22]. Since the hydrated form with acyclovir:water molar ratio 3:2 is used to produce solid drug products [21], the melting of API is preceded by dehydration occurring over a wide temperature range (~100 to 150 °C). It should be noted that the DSC curves of the three drug products are almost identical due to similar acyclovir content (Table 1, 75.8–79.1%) and the same excipients.

The DSC curves of the drug products Nifuroksazyd Hasco, Nifuroksazyd 200 Hasco, and Nifuroksazyd Richter are difficult to interpret (Figure 3, curves 15, 16, and 17, respectively). Although they contain nifuroxazide in an amount of 33–50% by the tablet mass (Table 1), the DSC curves of these products do not show a clear endothermic peak due to API melting but only a large exothermic peak above 267 °C. Taking into account the fact that, according to literature data, nifuroxazide melts at 282 °C [23], and its degradation, depending on the polymorphic form, begins in the range of 281–290 °C [24], degradation of the API occurs simultaneously with melting. Only in the case of Nifuroksazyd Richter tablets, a very flat endothermic effect preceding the exothermic effect is visible, which corresponds to the melting of the API. In contrast, the DSC curve of Paracetamol Polfa-Lodz tablets (Figure 2, curve 18) reveals that the peak due to the melting of paracetamol (Figure 2, curve 18a) is shifted toward lower values, to ~152 °C. Although the literature melting point of the polymorphic form I of paracetamol is 171 °C [15], its value may be lowered as a result of API amorphization under the influence of some excipients present in the studied tablets [34]. The melting of API is also preceded by an endothermic effect at ~100 °C, related to the melting of the γ form of sorbitol [35], which is also an ingredient of the tablet matrix.

A special case is the third group, which includes 16 drug products containing lactose monohydrate (Figure 4 and Figure 5) as the dominant excipient. Due to the intensity of the two endothermic DSC peaks associated with the dehydration of lactose monohydrate at ~140 °C and the subsequent melting of anhydrous α-lactose at ~220 °C [36], the interpretation of the DSC curves of these drug products is presented separately.

In the DSC curves of the drug products Furosemide Polpharma (Figure 4, curve 19) and Furosemidum Polfarmex (Figure 4, curve 20), the endothermic peak due to the melting of furosemide (Figure 4, curve 20a) [25] is superimposed on the melting peak of dehydrated lactose. An exothermic peak at ~220 °C is also visible, reflecting the degradation of furosemide. In the case of drug products Encorton 5 mg, 10 mg, and 20 mg (Figure 5, curves 21, 22, and 23, respectively), three endothermic DSC peaks are visible, with the last one not completely separated from the melting peak of dehydrated lactose and having an irregular shape. The literature melting point of prednisone is 235 °C [26,27]. The first two peaks reflect the dehydration and melting of lactose. Considering the very low content of prednisone in the tablets studied (Table 1, 4.2–8.4%), it can be assumed that the very small peak due to API melting is shifted toward lower temperatures and overlaps with the peak reflecting the melting of dehydrated lactose. However, the exothermic peak above 260 °C reflecting the degradation of prednisone is not visible.

On the other hand, in the DSC curves of Spironol 25 (Figure 5, curve 24) and Spironol 100 tablets (Figure 5, curve 25), a small endothermic peak at ~200 °C reflecting the melting of spironolactone [28] precedes a large endothermic peak due to the melting of dehydrated lactose. However, it is difficult to precisely separate the two peaks, preventing a more detailed interpretation.

The DSC curve of Metoclopramidum tablets (Figure 5, curve 26) shows several overlapping, weak endothermic peaks below 100 °C. Literature data suggest that one of these peaks is associated with dehydration of the API [29], which then amorphizes. Due to the low API content by the tablet mass (Table 1, 9.9%), the intensity of this peak is low. Above 105 °C, recrystallization of the API occurs, which should be confirmed by an exothermic peak—however, it is not visible on the DSC curve of the drug product. Instead, the peak associated with lactose dehydration is clearly marked. Above 185 °C, there are two overlapping endothermic peaks. The first peak corresponds to the melting of the crystalline form of metoclopramide hydrochloride, and the second to the melting of anhydrous lactose. However, above 250 °C, a broad exothermic peak is present due to API degradation [37].

The DSC curves of drug products Enarenal 5, 10, and 20 (Figure 5, curves 27, 28, and 29, respectively) look very similar, as they are virtually the same in terms of enalapril maleate content (Table 1, 7.9–8.0%) and tablet matrix composition (type and content of excipients). Similarly, as with most drug products containing lactose monohydrate, the endothermic DSC peak expected at ~143 °C due to melting of enalapril maleate [30] overlaps with the peak, confirming dehydration of this excipient.

The drug product Etopiryna (Figure 4, curve 30) contains three APIs. Acetylsalicylic acid is present in the largest amount (Figure 4, curve 30a, Table 1, 50.9% by the tablet mass), with ethenzamide (Figure 4, curve 30b, 17.0%) and caffeine (Figure 4, curve 30c, 8.5%) in smaller amounts. The Etopiryna DSC curve shows two overlapping endothermic peaks above 70 °C. The first peak is related to the melting of the eutectic mixture formed by acetylsalicylic acid and caffeine [20], while the second reflects the dehydration of lactose monohydrate. Above 200 °C, two more endothermic peaks are evident. The small and sharply ended peak is related to the melting of anhydrous lactose. In contrast, it is difficult to identify the origin of the second, very broad peak occurring immediately after it. Moreover, the characteristic peak due to the melting of ethenzamide at 132 °C is missing [38].

In the DSC curves of the drug products Tialorid (Figure 4, curve 31) and Tialorid mite (Figure 4, curve 32) containing hydrochlorothiazide (Figure 4, curve 32a) and amiloride hydrochloride, the peaks due to melting of hydrochlorothiazide and anhydrous lactose overlap to form a set of endothermic peaks over a wide temperature range (190–260 °C). It should be noted that the literature melting point of hydrochlorothiazide is slightly higher at 273 °C [31]. The area of the peak complex is larger for Tialorid tablets, as they contain twice the amount of hydrochlorothiazide per tablet mass (Table 1, 20.3%). However, at ~140 °C, a peak due to the dehydration of lactose monohydrate is visible, with its area also greater for Tialorid tablets. Moreover, due to the low content of amiloride hydrochloride in both drug products (~1–2%), a peak that reflects the melting of this API is not visible. The literature melting point of amiloride hydrochloride is 293 °C, and dehydration of the API occurs at ~132 °C [39].

The exceptions in the group of drug products containing lactose monohydrate as an excipient are Luminalum Unia 15 (Figure 5, curve 33) and Luminalum Unia 100 (Figure 5, curve 34) tablets. The endothermic peak due to phenobarbital melting (~173 °C) is sharply ended and clearly separated from the peaks associated with lactose dehydration and melting. The melting point of phenobarbital is consistent with literature data (~175 °C) [32]. In addition, because the phenobarbital content of Luminalum Unia 100 tablets is more than three times higher (Table 1, 53.1%), the peak on the DSC curve of Luminalum Unia 100 tablets reflecting API melting is also much larger.

### 2.2. Effect of Storage on Commercial Drug Products

With the aim of assessing the suitability of DSC as a method to identify adverse physicochemical changes occurring in commercial drug products during storage, six series of studies were performed between 2011 and 2022. The DSC analysis showed that the key phase transformation detected in the majority of the studied solid drug products was the API melting. At high API content, an endothermic peak due to melting was characterized by a large area and took place over a narrow temperature range. In contrast, it was very difficult to detect at low API content. In turn, no DSC effects that reflect polymorphic transformations were detected. On the other side, chemical processes occurring in some drug products were related to thermal degradation of APIs and were accompanied by exothermic DSC peaks.

The DSC curves of the studied drug products obtained during the first series of experiments (Figure 1, Figure 2, Figure 3, Figure 4 and Figure 5, February 2011) indicated some differences between the first two groups of drug products and group three. The differences are due to the low content of APIs in most of the drug products of the third group and, consequently, the high content of lactose monohydrate as an excipient. The data in Table 1 show that of the 16 drug products of group three, only two contain APIs in an amount slightly exceeding 50% of the tablet mass, while in eight products, the content of APIs does not exceed 10% of the tablet mass. This is crucial because the DSC curves of some of the tablets studied do not show the endothermic effect due to API melting.

The DSC curves also showed that, with the exception of three drug products containing nifuroxazide as an API (Nifuroksazyd Hasco, Nifuroksazyd 200 Hasco, and Nifuroksazyd Richter), endothermic DSC peaks due to melting of APIs appear for most of the tablets studied. In contrast, for 10 drug products in group three (Furosemidum Polpharma, Furosemidum Polfarmex, Encorton 5 mg, 10 mg and 20 mg, Enarenal 5, 10 and 20, Tialorid, and Tialorid mite), the melting ranges of APIs and dehydrated lactose overlap, forming a common, extended endothermic peak. Regardless, all tablets in group three are characterized by the presence of a large endothermic DSC peak due to dehydration of lactose monohydrate. In contrast, two drug products, Coffepirine and Etopiryna, behave differently—an endothermic DSC peak appears, reflecting melting of the eutectic mixture.

**Figure 6:**
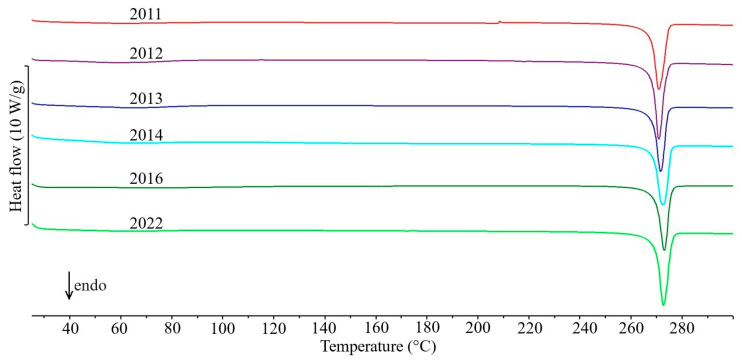
DSC curves of Theospirex retard 150 tablets made during six series of measurements conducted between 2011 and 2022.

**Figure 7:**
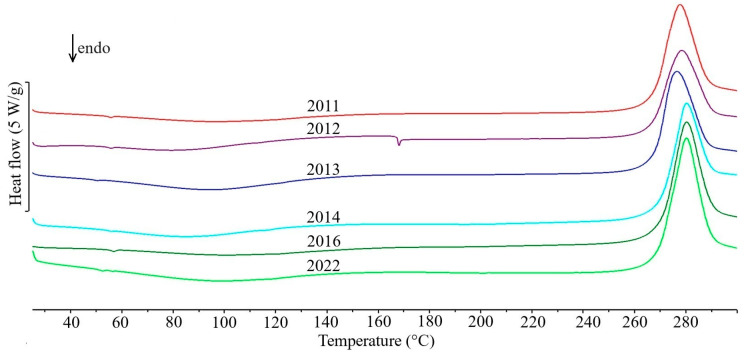
DSC curves of Nifuroksazyd Hasco tablets made during six series of measurements conducted between 2011 and 2022.

**Figure 8:**
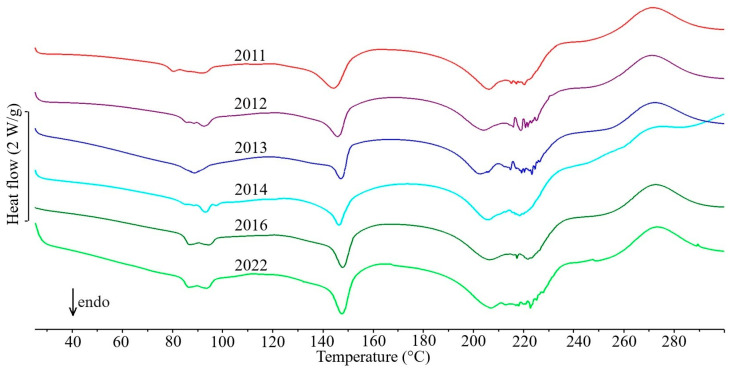
DSC curves of Metoclopramidum tablets made during six series of measurements conducted between 2011 and 2022.

Figure 6, Figure 7 and Figure 8 show the DSC curves for selected drug products recorded in six consecutive measurement series from 2011 to 2022, while the data for all series of measurements are summarized in Tables S1–S6 and in Table 1 for selected series. Visual analysis of the temperature ranges and shape of the endothermic DSC peaks, as well as the exothermic peak for drug products containing nifuroxazide, did not reveal any significant differences between the consecutive series of measurements during the study period for all drug products. Similarly, a comparison of the precision (standard deviations, SD) with which the characteristic peak temperatures and enthalpies of transition were determined for all peaks on the DSC curves of the studied drug products did not reveal significant differences between successive series of measurements performed between 2011 and 2022. It should be noted, however, that the precision of enthalpy determination was generally lower than the precision of transition temperature determination. Furthermore, as expected, the highest standard deviation values were observed for exothermic DSC peaks associated with the thermal decomposition of the studied drug products.

Therefore, considering that the beginning of API degradation and the appearance of impurities in the drug product affect the peak onset temperature (*T_on_*) and peak temperature (*T_p_*), as well as the value of the enthalpy of melting (Δ*H_f_*) and the shape of the endothermic DSC peak due to melting [40,41], it was decided to use these parameters to detect the first changes indicating undesirable processes in commercial drug products during long storage. This research confirmed that only a detailed analysis of the temperatures and enthalpies of phase transformations (*T_on_*, *T_p_*, and Δ*H_f_*) indicated slight differences between the data for successive series of measurements.

The values of the onset (*T_on_*) and peak (*T_p_*) temperatures for DSC peaks should be determined with the understanding that, in the case of a melting transition, the only thermodynamically significant parameter is *T_on_*, which corresponds to the real melting temperature of the substance under study. It is determined as the point where the pre-melting baseline intersects with the leading edge of the DSC peak. It is independent of measurement conditions, such as heating rate, sample mass, measuring instrument, and others. In contrast to *T_on_*, *T_p_* is the temperature at which the examined substance is fully melted. Since its value depends on all the aforementioned measurement parameters, *T_p_* is not a characteristic property of the substance under study. For example, as the sample mass increases, *T_p_* shifts toward higher temperatures due to the finite time required for heat transfer within the sample. This means that *T_p_* is not a physical value representative of the melting point and therefore should not be used to compare results for different products containing varying amounts of the same API, obtained under different experimental conditions, using different instruments, or in different laboratories, as *T_p_* values will not match, which may lead to erroneous conclusions.

As expected, the values of *T_on_* and *T_p_* should have shifted toward lower values in the event that the APIs studied degraded during long storage. However, no such trend was observed in every situation. The *T_on_* and *T_p_* shifted slightly toward lower or higher temperatures, with greater changes recorded for *T_on_* than for *T_p_*. In general, *T_on_* changed between 1 and 5 °C between 2011 and 2022, while *T_p_* only changed between 1 and 2 °C. The peak area (Δ*H_f_*) also changed slightly, decreasing for most DSC peaks. The decrease in *T_on_* and *T_p_* values and the broadening of the melting range can be explained by the presence of API degradation products, where the relationship between the decrease in melting point due to the presence of impurities and their concentration is quantitatively described by the van’t Hoff equation [9]. In turn, the shift of *T_on_* and *T_p_* towards higher values can be explained based on the results of research on the drug substance piracetam stored at 60 °C for 91 days [42]. It has been shown that the purity of the API increases during storage because the rate of decomposition of intrinsic impurities embedded in the piracetam structure is greater than the rate of decomposition of the API. However, without additional evidence, this explanation cannot be applied to other APIs. Alternatively, the increased thermal stability can be explained by the crystallization of the amorphous API over time.

Considering that the melting enthalpy (Δ*H_f_*) determined from DSC curves is a thermodynamic quantity that defines the amount of energy exchanged with surroundings in the form of heat by the API in its crystalline form, proportional to the amount of API in the dosage form, it was decided to use experimentally determined Δ*H_f_* values to calculate the API content in the drug products under study in successive measurement series, with the aim of confirming whether this method can be used to monitor the decreasing quality of the tablets resulting from the decrease in the amount of API within them. Using data declared by manufacturers on the API contents in the studied tablets (Table 1), the Δ*H_f_* values for the APIs used as standards, and the Δ*H_f_* values determined for the studied tablets (Tables S1–S6), the API content in commercial drug products was calculated in successive measurement series (2011–2022), and the results are summarized in Table 2.

When interpreting the results shown in Table 2, it is important to consider that commercial pharmaceutical tablets are products with specific characteristics; that is, they are multi-component mixtures manufactured using various technological processes, including the application of heat, organic solvents, and high tablet press stamping force, ranging from several to several dozen kN. Therefore, a simple comparison of the melting enthalpy of the API as a standard with the melting enthalpy of the API in the drug product may lead to significant errors, resulting, among other things, from the fact that the influence of excipients on the API melting process is not taken into account in the calculations. In particular, one should take into account the overlap of peaks with the melting peak of the API, especially peaks resulting from slight sublimation, evaporation, or decomposition of the melted API [13]. Interactions between the API and selected excipients, leading to amorphization or the formation of solid dispersions with the API, can also significantly affect the results of the determinations.

For the above reasons, the results summarized in Table 2 should be considered preliminary (indicative). Only in the case of tablets of Theospirex retard 150, Theospirex retard 300, Pyralgina, Ranigast Max, Cyclonamine, Heviran 200, and Luminalum Unia 100 are the results of API content determinations in the studied drug products from the first series of experiments (in February 2011) consistent with the values declared by the manufacturers, i.e., they fall within the range of mean ± 5% [5]. In other cases, the results are generally lower than the values declared by the manufacturers, which may be caused, among other things, by the amorphization of APIs by polymeric excipients [34]. Conversely, API content higher than declared was found for Heviran 400, Heviran 800, and Metoclopramidum tablets. This is likely due to the partial overlap of the DSC peaks of the excipients with the API melting peak. A special case is Metoclopramidum tablets, in which the content of metoclopramide hydrochloride was found to be 15 times higher than the value declared by the manufacturer. This result may be attributed to the complexity of the processes occurring during API heating, i.e., dehydration, recrystallization of the amorphous substance, and melting of the recrystallized API, which are partially overlapped by the melting of anhydrous lactose. The low content of metoclopramide hydrochloride in the tablets (Table 1, 9.9%) may also be a contributing factor. An analysis of the results from subsequent measurement series confirmed that the API content in the studied drug products is generally lower than the values obtained in the first measurement series.

The data obtained suggest that further research is needed to develop reliable quantitative analysis methods based on calibration curves of the melting enthalpy (Δ*H_f_*) as a function of API concentration in the tablet matrix, using deconvolution of the DSC peak related to API melting from the partially overlapping peaks of the excipients [13]. For this reason, and considering the large measurement error for Metoclopramidum tablets, the API content was not determined in the following drug products: Cipronex 250 and Cipronex 500 (a very weak endothermic peak due to API melting, Δ*H_f_*~10 J/g); Nifuroxazide Hasco, Nifuroxazide 200 Hasco, and Nifuroxazide Richter (no endothermic peak due to API melting); Furosemide Polpharma, Furosemide Polfarmex, Encorton 5 mg, Encorton 10 mg, Encorton 20 mg, Spironol 25, Spironol 100, Tialorid, and Tialorid mite (partial or complete overlap of DSC endothermic peaks due to melting of API and dehydrated lactose); Enarenal 5, Enarenal 10, and Enarenal 20 (API melting and lactose monohydrate dehydration occur within the same temperature range); and Coffepirine and Etopiryna (interaction between components, eutectic formation).

It is not possible to compare the observed changes in *T_on_* and Δ*H_f_* values, as well as in API contents of the studied drug products, with literature data, since DSC studies of commercial solid drug products stored for several years under conditions recommended by the European Pharmacopoeia (Ph. Eur.) are absent in the world literature. This is further complicated by the complex matrix composition of the tablets studied. As mentioned above, in addition to APIs, tablets contain from three to six excipients, with coated tablets characterized by an even more complex composition due to a tablet core coating composed of several excipients. As is well known, excipients can affect the stability of APIs, as confirmed by the few thermal studies of commercially available solid drug products. For instance, DSC curves of omeprazole in bulk alone and omeprazole in granulated form were found to differ [43]. The reason for this is presumably the steric impedance generated by the polymeric excipient and the formation of hydrogen bonds between the API and the polymer, limiting the formation of intermolecular bonds between the APIs. In turn, a study of two different commercial tablets with prednisone showed that the DSC curves of both drug products are very similar to the curve of lactose, because the lactose content in tablets is almost three times higher than that of prednisone [44]. Moreover, the rate constants of thermal degradation by isothermal thermogravimetry (isothermal TG) showed that prednisone in bulk alone is more stable than the tablets. A study of the thermal degradation of cimetidine led to the opposite conclusion; this API is more stable in tablet form than in bulk alone [45]. The effect of talc concentration on the expiration date of acetaminophen (paracetamol) tablets was also studied using TG [46]. It has been found that talc does not affect the degradation mechanism of the API, but it enhances the API degradation rate.

### 2.3. Interpretation of the DSC Data Using PCA

The study showed that the comparison of temperatures and enthalpies of phase transformations recorded in six consecutive measurement series from 2011 to 2022 does not provide reliable data to detect changes in physical transformations and chemical processes in commercial drug products during storage. Therefore, an advanced method of statistical analysis, principal component analysis (PCA), was used to interpret the data obtained.

Using mathematical computations, PCA transforms multivariate data matrices with correlated variables (heat flow values acquired from DSC curves every 0.17 °C, in the range of 25–300 °C, for six study series from 2011–2022), into new matrices with uncorrelated variables (orthogonal, independent of each other), called principal components (PCs) [47]. The new PCs describe the variability in the data matrix. The first principal component (PC1) reflects most of the variability in the data matrix, while the second (PC2) accounts for the next highest variability. PCA computations showed that PC1 describes between 71.42% (Heviran 200) and 97.56% (Spironol 100) of the variability in the 34 matrices studied, while PC2 only describes between 1.69% (Spironol 100) and 23.87% (Cipronex 500) of the variability. Therefore, the results of the PCA are presented in two-dimensional score scatter plots, PC1 and PC2.

**Figure 9:**
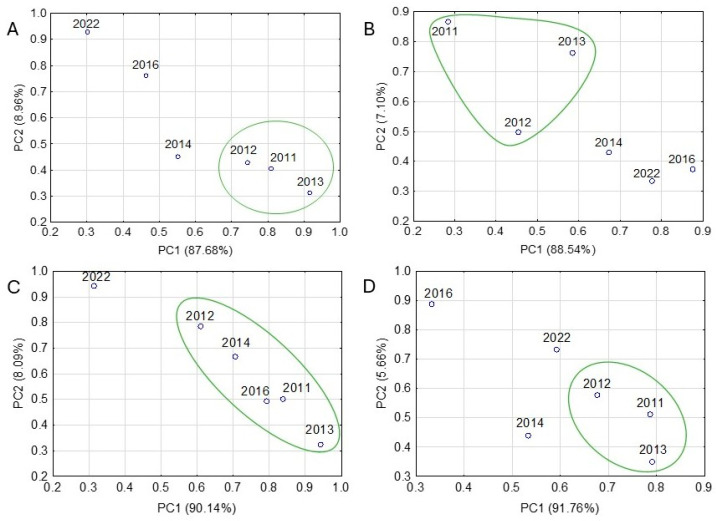
PCA score scatter plots for tablets of Theospirex retard 300 (**A**), Heviran 800 (**B**), Cipronex 250 (**C**), and Luminalum Unia 100 (**D**), based on the DSC data acquired during six series of measurements from February 2011 to July 2022.

Figure 9 shows selected examples of DSC data interpretation using PCA. A detailed inspection of these examples reveals that the points on the scatter plots of the PCA results, which reflect the DSC curves for successive series of measurements, vary in location. This indicates that the DSC curves differ slightly from each other and thus reflect minor physicochemical changes in drug products during long-term storage. The results of the study, therefore, indicate that only the use of PCA makes it possible to separate the DSC curves in such a way that the first signs of deterioration can be detected. Probably, they are related to the beginning of API degradation, and the resulting degradation products affect the parameters of the DSC curves.

The distinctive location of points on the PCA plots (Figure 9) inspired a challenge to see if chemometrically processed DSC data would distinguish drug products before their expiration date from out-of-date products. The PCA plot of Theospirex retard 300 tablets (Figure 9A) shows that for all drug products in group one, clusters are formed in the narrow range of PC1 and PC2 values (lower right corner of the plot), grouping the DSC data for the first three series of studies (February 2011, March 2012, and September 2013). The location in a common cluster of the results obtained before the expiration date of drug products indicates that the DSC curves recorded between 2011 and 2013 are almost identical. This is evidence that no undesirable physical transformations and chemical processes have occurred in the drug products studied. On the other hand, the DSC data obtained after the expiration date (consecutive series of studies: December 2014, November 2016, and July 2022) are located in the PCA plots at different places, more or less distant from the cluster grouping the data for the first three series. This indicates that these curves differ from each other and from the curves for the first three series of measurements, suggesting that undesirable physicochemical transformations may have occurred in drug products.

The results of PCA computations indicate that the clear separation of DSC curves obtained before the expiration date, from those recorded after the expiration date, is conditioned by the high content of APIs in the examined drug products (from 82.3 to 91.7% of the tablet mass). For this reason, the low content of excipients in drug products of this group (from ~8 to ~18%) is unable to significantly affect the phase transformation of APIs and the shape of DSC peaks. This allows DSC to be used to detect the first changes in physical transformations and chemical processes that have occurred in drug products.

Of the drug products in group two, only in seven of them—Ranigast Max, Cyclonamine, Coffepirine, Heviran 800, Nifuroksazyd Hasco, Nifuroksazyd 200 Hasco, and Paracetamol Polfa-Lodz—did PCA computations indicate a clear separation between DSC data obtained before the expiration date of the tablets and those obtained after that date. This is illustrated in Figure 9B using the example of Heviran 800 tablets, which contain acyclovir at 75.8% of the tablet mass. The DSC data for the first three series of studies (February 2011, March 2012, and September 2013) form a common cluster in the upper left corner of the PC1 and PC2 plot. In the opposite corner of the plot are data obtained after the product’s expiration date.

For other drug products in group two, PCA results are not satisfactory. An example is Cipronex 250 tablets (Figure 9C). Despite the fact that they contain ciprofloxacin hydrochloride as an API at 62.2% by the tablet mass, it is difficult to logically justify the location on the PCA plot of the DSC data for the following six study series. In a line from the lower right corner of the plot to the center of the plot, DSC data obtained in September 2013, February 2011, November 2016, December 2014, and March 2012 are grouped together, suggesting a relatively high similarity between the 2011–2016 DSC curves and the absence of changes in physicochemical transformations in the Cipronex 250 tablets despite the 6-year storage period. This is unrealistic and difficult to substantiate.

It should be mentioned that all drug products for which satisfactory results were not obtained are coated tablets. They are characterized by a very complex composition of the tablet matrix. They differ from the first group of tablets in that their core, which usually consists of 3–5 excipients, is covered by a layer of an envelope composed of several ingredients.

For the third group, PCA plots make it possible to separate DSC data obtained before the expiration date from data obtained after that date, for 11 drug products: Furosemidum Polpharma, Furosemidum Polfarmex, Encorton 5 mg, Encorton 10 mg, Encorton 20 mg, Spironol 100, Enarenal 20, Etopiryna, Tialorid, Luminalum Unia 15, and Luminalum Unia 100. This is shown in Figure 9D using the example of Luminalum Unia 100 tablets, which contain phenobarbital at 53.1% by the tablet mass. Among these drug products, the exceptions are Encorton 5 mg, Encorton 10 mg, Encorton 20 mg, and Enarenal 20 tablets, in which the content of APIs ranges from 4.2 to 8.4% of the tablet mass, while the melting ranges of APIs and dehydrated lactose overlap, forming a common extended endothermic DSC peak. Therefore, it can be assumed that the crucial influence on the PCA results for these tablets was excipients (in particular lactose monohydrate), present in these drug products in the amount of ~91–95% of the tablet mass.

Unsatisfactory results were obtained for those drug products in group three, where the API content was less than 10%. The reason for this is probably the high content of various excipients, including lactose monohydrate. By undergoing various physicochemical transformations during long storage, excipients affect the shape of drug products’ DSC curves by distorting subtle effects caused by the presence of APIs. This leads to false results from the point of view of detecting the changes in physical transformations and chemical processes in APIs contained in these drug products. The case of Spironol 25 tablets, which contain APIs in the amount of 20.6% of the tablet mass, can be explained in a similar way.

## 3. Materials and Methods

### 3.1. Chemicals

Thirty-four solid drug products available in pharmacies in Poland were examined, as listed in Table 1. They contained 19 different APIs, ranging from 1 to 92% by the tablet mass. The tablets were as follows: Cipronex 250, Cipronex 500, Enarenal 5, Enarenal 10, Enarenal 20, Etopiryna, Furosemidum Polpharma, Heviran 200, Heviran 400, Heviran 800, Metoclopramidum, Pyralgina, Ranigast Max, Ranigast Polpharma, Tialorid mite, Tialorid (Polpharma, Starogard Gdanski, Poland); Coffepirine (Marcmed, Lublin, Poland); Cyclonamine (Galena, Wroclaw, Poland); Encorton 5 mg, Encorton 10 mg, Encorton 20 mg (Polfa, Pabianice, Poland); Furosemidum Polfarmex (Polfarmex, Kutno, Poland); Luminalum Unia 15, Luminalum Unia 100 (Unia, Warszawa, Poland); Nifuroksazyd Hasco, Nifuroksazyd 200 Hasco (Hasco-Lek, Wroclaw, Poland); Nifuroksazyd Richter, Spironol 25, Spironol 100 (Polfa, Grodzisk Mazowiecki, Poland); Paracetamol Aflofarm (Aflofarm, Ksawerow, Poland); Paracetamol Biofarm, Theospirex retard 150, Theospirex retard 300 (Biofarm, Poznan, Poland); and Paracetamol Polfa-Lodz (Polfa-Lodz, Lodz, Poland).

The following APIs were also examined: acetylsalicylic acid, ethenzamide, ranitidine hydrochloride (Polpharma, Starogard Gdanski, Poland); caffeine, furosemide, hydrochlorothiazide, paracetamol, theophylline (Sigma-Aldrich, Steinheim, Germany); and metamizole sodium (Galfarm, Krakow, Poland). The purity of all APIs was ≥99%.

### 3.2. Sample Preparation

The studied drug products in their original packaging were stored in a drug warehouse under the conditions recommended by the European Pharmacopoeia (Ph. Eur.), i.e., at a temperature range of 15–25 °C, ≤60% of relative humidity, and in a location protected from direct sunlight [5]. At appropriate intervals, i.e., in February 2011, March 2012, September 2013, December 2014, November 2016, and July 2022, three tablets were randomly selected from each package and, after appropriate preparation, analyzed using the DSC method. The remaining tablets in the packages were stored in the drug warehouse until the next DSC study.

To prepare samples for DSC studies and determine the average tablet mass, three tablets randomly taken from each package were weighed on a WAA 100/X/1 analytical balance (Radwag, Radom, Poland). The three tablets were then gently crushed together in a mortar using a pestle, avoiding the high pressure of the pestle to prevent possible interactions between the tablet ingredients. The powdered material was examined using DSC.

Six series of DSC studies were carried out. The first three series were performed in 2011–2013 (in February 2011, March 2012, and September 2013). Since, according to the manufacturers’ declarations, the expiration dates of the examined drug products ended in 2013, in order to detect unfavorable changes in these products after the expiration date, three more series of DSC studies were performed between 2014 and 2022 (in December 2014, November 2016, and July 2022).

### 3.3. DSC Measurements

Samples of 3.90–4.10 mg were weighed in 40 µL aluminum containers using a Mettler Toledo Dual Range XA 105 balance (Schwerzenbach, Switzerland). Each container was sealed with a lid with two holes. Experiments were carried out using a heat-flux DSC 822e (Mettler Toledo, Schwerzenbach, Switzerland) with a liquid nitrogen cooling system (Dewar vessel). Samples were heated in the temperature range of 25–300 °C at a rate of 10 °C/min in a nitrogen atmosphere flowing at a rate of 70 mL/min. From the DSC curves, the phase transition onset temperatures (*T_on_*), peak temperatures (*T_p_*), and enthalpies of melting (Δ*H_f_*) were determined using STARe 9.10 and STARe 15.0 software. All DSC measurements were performed in triplicate, and the results are presented as the mean ± standard deviation (SD).

The DSC apparatus was calibrated before each series of measurements with high-purity metals, indium (In, purity 99.999%) and zinc (Zn, purity 99.998%) (Mettler Toledo, Schwerzenbach, Switzerland). The *T_on_* and *∆H_f_* reference values with the tolerance limits were for indium 156.6 ± 0.3 °C and 28.45 ± 0.6 J/g, and for zinc 419.6 ± 0.7 °C and 107.5 ± 3.2 J/g. The values obtained in triplicate during calibration were as follows: 156.63 ± 0.12 °C and 28.47 ± 0.24 J/g for the former, and 419.62 ± 0.14 °C and 107.45 ± 0.78 J/g for the latter. Calibration and all the necessary adjustments were performed with the aid of the computer program Calib DSC Total In/Zn (Mettler Toledo, Schwerzenbach, Switzerland).

### 3.4. PCA Computations

Statistica 13.3 software (TIBCO Software Inc., Paolo Alto, CA, USA) was used for principal component analysis (PCA) computations. Thirty-four matrices were created individually for each of the studied drug products listed in Table 1. The variables in these matrices were heat flow values (W/g), determined from DSC curves every 0.17 °C over the temperature range of 25–300 °C. The PCA computations used DSC curves recorded for six consecutive series of measurements carried out between 2011 and 2022. The correlation matrix and varimax rotation algorithm were used for PCA computations.

## 4. Conclusions

The study of 34 commercial solid drug products, stored under conditions recommended by the Ph. Eur. for a long period of time (2011–2022), showed that the key phase transition detected in most samples was API melting. The endothermic peaks associated with melting were characterized by a large area and occurred over a narrow temperature range only in the case of high API content in drug products. No DSC peaks reflecting polymorphic transformations were detected, while exothermic DSC effects for some drug products indicated degradation of the API.

Visual analysis of the temperature ranges and shape of the endothermic peaks for all drug products showed no significant differences between the DSC curves registered for six consecutive series of measurements. Satisfactory results were also not obtained for the determination of API content in the drug products studied due to the significant influence of excipients on the Δ*H_f_* values for API melting. Only the use of PCA for detailed analysis of the temperatures and enthalpies of phase transformations (*T_on_*, *T_p_*, and Δ*H_f_*) proved that the particular DSC curves differ slightly from each other and thus reflect minor physicochemical changes in drug products during long-term storage. This makes it possible to detect the first signs of deterioration, probably related to the appearance of API degradation products.

An undoubted benefit of the study may be the use of chemometrically processed DSC data to distinguish drug products before the expiration date (quality guaranteed by manufacturers) from drug products after the expiration date. This is possible in more than half of the drug products studied.

The possibility of obtaining satisfactory results is determined by the content ratio of API to excipients in the tablet and the complex composition of the tablet matrix. The higher it is, i.e., the higher the API content and the lower the excipients, the higher the probability of detecting the first signs of API deterioration in a drug product. The PCA can be especially helpful in this regard.

## Figures and Tables

**Table 1:** Characterization of studied commercial solid drug products and data for selected series of DSC measurements.

No	Trade Names of Drug Products	Active Pharmaceutical Ingredients			DSC Peaks Related to the Melting of Active Pharmaceutical Ingredients Contained in Drug Products								
		Dominant API	Content (%) *	Melting Point (°C)	February 2011			September 2013			July 2022		
					*T_on_* (°C)	*T_p_* (°C)	Δ*H_f_* (J/g)	*T_on_* (°C)	*T_p_* (°C)	Δ*H_f_* (J/g)	*T_on_* (°C)	*T_p_* (°C)	Δ*H_f_* (J/g)
1	Theospirex retard 150 ^3^	theophylline	82.3	272 [14]	267.6	269.7	126.1	268.1	270.4	112.2	269.2	271.7	119.0
2	Theospirex retard 300 ^3^		83.3		267.6	270.0	125.6	266.3	269.5	124.2	269.9	272.4	126.0
3	Paracetamol Biofarm ^1^	paracetamol	89.4	171 [15]	167.3	170.5	153.9	167.5	170.1	146.8	168.3	171.9	153.7
4	Paracetamol Aflofarm ^1^		91.7		167.1	170.7	146.9	167.6	170.8	149.4	168.5	170.7	149.3
5	Pyralgina ^1^	metamizole sodium	86.1	216 [16]	216.9	228.8	44.2	217.2	223.0	44.6	218.7	224.2	39.6
6	Ranigast Polpharma ^2^	ranitidine hydrochloride	54.6	151 [17]	142.2	146.5	54.9	141.8	146.1	54.1	144.1	147.9	56.3
7	Ranigast Max ^2^		54.1		142.6	146.9	62.3	143.6	147.4	60.0	143.4	147.6	61.2
8	Cyclonamine ^1^	etamsylate	80.6	137 [18]	127.3	129.4	108.1	127.9	129.4	108.6	128.3	130.5	110.3
9	Cipronex 250 ^2^	ciprofloxacin hydrochloride	62.2	303 [19]	280.0	288.3	11.3	280.2	286.9	9.24	281.6	287.5	11.7
10	Cipronex 500 ^2^		65.3		283.3	289.1	10.2	281.2	286.4	11.1	281.2	286.2	11.9
11	Coffepirine ^1^	acetylsalicylic acid ^a^	81.9	137 [20] **	114.9	127.0	138.7	112.2	125.9	142.0	116.4	128.1	116.4
12	Heviran 200 ^2^	acyclovir	79.1	254 [21], 255 [22]	246.0	250.0	96.6	246.0	249.2	95.7	247.2	251.3	97.5
13	Heviran 400 ^2^		77.0		248.2	250.5	97.5	249.23	251.6	93.5	249.4	252.1	95.2
14	Heviran 800 ^2^		75.8		246.5	250.2	100.8	247.4	250.3	96.7	247.9	251.7	100.3
15	Nifuroksazyd Hasco ^2^	nifuroxazide	49.7	282 [23], 281–290 [24] ***	267.7	278.8	301.3	268.1	277.2	289.1	270.7	280.9	320.4
16	Nifuroksazyd 200 Hasco ^2^		48.3		268.6	278.0	318.1	268.7	278.2	275.6	270.0	280.9	326.9
17	Nifuroksazyd Richter ^2^		32.9		270.9	278.0	287.8	270.8	278.6	253.1	271.3	278.3	303.1
18	Paracetamol Polfa-Łódź ^1^	paracetamol	63.0	171 [15]	151.8	159.4	107.7	151.9	159.4	98.2	152.6	159.6	101.8
19	Furosemidum Polpharma ^1^	furosemide	42.1	203 [25]	197.9	208.9	77.7	198.4	209.6	71.0	201.8	211.6	85.7
20	Furosemidum Polfarmex ^1^		40.5		195.5	206.8	72.9	192.6	208.1	71.0	197.6	207.9	80.3
21	Encorton 5 mg ^1^	prednisone	4.2	235 [26,27]	203.6	211.7	91.4	205.2	212.0	82.6	205.4	213.4	94.4
22	Encorton 10 mg ^1^		4.2		204.5	212.1	90.3	205.2	212.5	76.8	205.8	213.4	94.8
23	Encorton 20 mg ^1^		8.4		204.2	212.0	93.8	204.8	212.2	77.8	205.7	213.3	90.0
24	Spironol 25 ^1^	spironolactone	20.6	208 [28]	200.4	210.4	70.9	199.2	209.8	69.3	203.1	213.2	67.3
25	Spironol 100 ^2^		20.6		198.4	209.2	66.6	197.9	208.7	65.8	200.6	211.8	63.2
26	Metoclopramidum ^1^	metoclopramide hydrochloride	9.9	184 [29]	187.9	206.1	143.2	188.2	222.9	129.8	188.3	216.2	139.7
27	Enarenal 5 ^1^	enalapril maleate	8.0	143 [30]	135.1	145.9	110.7	138.8	148.0	111.6	138.1	147.2	110.7
28	Enarenal 10 ^1^		7.9		136.3	144.8	93.0	137.5	147.0	91.9	138.7	147.1	92.9
29	Enarenal 20 ^1^		7.9		138.1	147.1	104.9	139.8	148.4	104.9	140.2	148.8	103.4
30	Etopiryna ^1^	acetylsalicylic acid ^a,b^	50.9	137 [20] **	72.0	78.7	30.53	68.0	76.3	32.9	71.1	78.4	27.1
31	Tialorid ^1^	hydrochlorothiazide ^c^	20.3	273 [31]	199.0	208.1	79.8	197.7	209.0	72.8	201.0	209.8	75.0
32	Tialorid mite ^1^		9.9		201.6	207.6	179.6	201.6	208.1	169.2	202.8	209.3	171.6
33	Luminalum Unia 15 ^1^	phenobarbital	15.0	175 [32]	169.1	171.7	14.5	169.2	171.8	13.9	170.3	173.0	15.0
34	Luminalum Unia 100 ^1^		53.1		170.0	173.2	69.2	170.1	172.9	59.7	171.1	174.1	59.0

^1^—uncoated tablets; ^2^—coated tablets; ^3^—prolonged release tablets; additional APIs are included in the drug products: ^a^—caffeine; ^b^—ethenzamide; ^c^—amiloride hydrochloride; *—API content per tablet mass; **—acetylsalicylic acid and caffeine contained in Coffepirine and Etopiryna tablets form a eutectic with a melting point of 107 °C; ***—DSC curves of Nifuroksazyd Hasco, Nifuroksazyd 200 Hasco, and Nifuroksazyd Richter tablets lack the endothermic DSC peak reflecting the melting of nifuroxazide, but there is a large exothermic peak above 267 °C. Accordingly, the values of *T_on_*, *T_p_*, and Δ*H_f_* for the exothermic DSC peak are reported.

**Table 2:** The content of APIs in crystalline form in commercially available drug products, calculated based on the melting enthalpies determined from DSC curves in successive measurement series. Characterization of studied commercial solid drug products and data for selected series of DSC measurements.

No	Trade Names of Drug Products	Active Pharmaceutical Ingredients			Melting Enthalpies and API Content in Commercial Drug Products in Successive Measurement Series											
		Dominant API	Δ*H_f_* (J/g)	Content (%) *	February 2011		March 2012		September 2013		December 2014		November 2016		July 2022	
					Δ*H_f_* (J/g)	Content (%) **	Δ*H_f_* (J/g)	Content (%) **	Δ*H_f_* (J/g)	Content (%) **	Δ*H_f_* (J/g)	Content (%) **	Δ*H_f_* (J/g)	Content (%) **	Δ*H_f_* (J/g)	Content (%) **
1	Theospirex retard 150	theophylline	153.4	82.3	126.1	82.2	127.8	83.3	112.2	73.1	118.0	76.9	114.7	74.8	119.0	77.6
2	Theospirex retard 300			83.3	125.6	81.9	125.7	81.9	124.2	81.0	126.7	82.6	124.8	81.4	126.0	82.1
3	Paracetamol Biofarm	paracetamol	187.4	89.4	153.9	82.1	151.5	80.8	146.8	78.3	153.4	81.9	150.4	80.3	153.7	82.0
4	Paracetamol Aflofarm			91.7	146.9	78.4	144.5	77.1	149.4	79.7	149.5	79.8	145.7	77.7	149.3	79.7
5	Pyralgina	metamizole sodium	52.7	86.1	44.2	83.9	45.6	86.3	44.6	84.6	39.9	75.7	39.9	75.7	39.6	75.1
6	Ranigast Polpharma	ranitidine HCl	116.6	54.6	54.9	47.1	56.5	48.5	54.1	46.4	52.7	45.2	53.3	45.7	56.3	48.3
7	Ranigast Max			54.1	62.3	53.4	61.0	52.3	60.0	51.5	60.2	51.6	60.0	51.5	61.2	52.5
8	Cyclonamine	etamsylate	135.6	80.6	108.1	79.7	118.7	87.5	108.6	80.1	112.0	82.6	105.5	77.80	110.3	81.3
9	Heviran 200	acyclovir	119.8	79.1	96.6	80.6	95.7	79.9	95.7	79.7	92.6	77.3	95.3	79.5	97.5	81.4
10	Heviran 400			77.0	97.5	81.4	95.3	79.5	93.5	78.0	97.1	81.1	92.6	77.3	95.2	79.5
11	Heviran 800			75.8	100.8	84.1	99.5	83.1	96.7	80.7	98.1	81.9	100.4	83.8	100.3	83.7
12	Paracetamol Polfa-Łódź	paracetamol	187.4	63.0	107.7	57.5	97.4	52.0	98.2	52.4	100.1	53.4	101.2	54.0	101.8	54.3
13	Metoclopramidum	metoclopramide HCl	94.9	9.9	143.2	150.9	131.0	138.0	129.8	136.8	138.0	145.4	136.9	144.3	139.7	147.2
14	Luminalum Unia 15	phenobarbital	127.1	15.0	14.5	11.4	14.9	11.7	13.9	10.9	14.00	11.0	14.4	11.3	15.0	11.8
15	Luminalum Unia 100			53.1	69.2	54.4	58.7	46.2	59.7	47.0	60.6	47.7	57.9	45.6	59.0	46.4

*—API content per tablet mass declared by the manufacturers; **—API content per tablet mass calculated based on the melting enthalpies determined from endothermic DSC peaks.

## Data Availability

The original contributions presented in this study are included in the article and Supplementary Materials. Further inquiries can be directed to the corresponding author.

## Supplementary Materials

The following supporting information can be downloaded at: https://www.mdpi.com/article/10.3390/molecules31081280/s1, Table S1: Results of the DSC measurements for all drug products obtained during the first series of experiments (February 2011); Table S2: Results of the DSC measurements for all drug products obtained during the second series of experiments (March 2012); Table S3: Results of the DSC measurements for all drug products obtained during the third series of experiments (September 2013); Table S4: Results of the DSC measurements for all drug products obtained during the fourth series of experiments (December 2014); Table S5: Results of the DSC measurements for all drug products obtained during the fifth series of experiments (November 2016); Table S6: Results of the DSC measurements for all drug products obtained during the sixth series of experiments (July 2022).

## Abbreviations

The following abbreviations are used in this manuscript:

API	Active Pharmaceutical Ingredient
DSC	Differential Scanning Calorimetry
HPLC	High-Performance Liquid Chromatography
PCA	Principal Component Analysis
Ph. Eur.	European Pharmacopoeia
