# Supplementary material for: Detecting the Physicochemical Transformations in Solid Drug Products Stored for Long Periods of Time—Insights into DSC Application
Source: Molecules. 2026 Apr 14;31(8):1280. doi: 10.3390/molecules31081280 (PMC13118484; doi:10.3390/molecules31081280)
Supplement: Supplementary file 1 [file molecules-31-01280-s001.zip › molecules-4225650-supplementary.pdf]

Supplementary Materials

Detecting the Physicochemical Transformations in Solid Drug Products Stored for Long Periods of Time—Insights into DSC Application

Edyta Leyk, Tomasz Konarski and Marek Wesolowski *

Department of Analytical Chemistry, Medical University of Gdansk, Gen. J. Hallera 107, 80-416 Gdansk, Poland; edyta.leyk@gumed.edu.pl (E.L.); t.konarski24@gmail.com (T.K.)
* Correspondence: marek.wesolowski@gumed.edu.pl

Table S1. Results of the DSC measurements for all drug products obtained during the first series of experiments (February 2011).

No	Trade names of drug products	Peak number	Temperature and enthalpy values for phase transitions (mean ± standard deviations, n = 3)		
			February 2011		
			T_{on} (°C)	T_p (°C)	ΔH_f (J/g)
1	Theospirex retard 150	peak 1	267.56±0.40	269.73±0.36	126.11±4.82
2	Theospirex retard 300	peak 1	267.57±0.14	270.02±0.34	125.64±1.95
3	Paracetamol Biofarm	peak 1	167.28±0.27	170.47±0.43	153.88±10.53
4	Paracetamol Aflofarm	peak 1	167.13±0.25	170.65±0.76	146.89±3.29
5	Pyralgina	peak 1	105.01±3.95	129.45±4.00	139.02±5.95
		peak 2	216.85±0.35	228.78±0.35	44.24±1.36
		peak 3*	224.85±0.26	231.72±0.34	280.26±6.41
6	Ranigast Polpharma	peak 1	142.15±0.58	146.51±0.47	54.89±1.12
		peak 2*	149.13±0.58	153.59±0.48	294.21±9.76
7	Ranigast Max	peak 1	142.62±0.74	146.88±0.68	62.31±2.58
		peak 2*	149.52±0.95	153.46±1.25	295.49±4.77
8	Cyclonamine	peak 1	127.31±0.21	129.43±0.18	108.10±2.04
9	Cipronex 250	peak 1	124.77±2.42	144.30±0.40	82.49±10.30
		peak 2	280.03±2.08	288.31±3.25	11.28±1.14
10	Cipronex 500	peak 1	122.95±2.68	144.64±1.00	94.38±3.84
		peak 2	283.31±0.91	289.12±0.75	10.19±1.14
11	Coffepirine	peak 1	114.92±12.67	126.98±1.33	138.68±5.80
12	Heviran 200	peak 1	106.82±13.22	135.01±0.12	122.22±4.74
		peak 2	169.95±0.16	176.94±0.39	5.03±0.51
		peak 3	246.02±1.66	250.04±1.07	96.59±0.27

		peak 4*	253.62±1.38	255.75±1.35	15.33±1.05
13	Heviran 400	peak 1	103.32±1.60	130.22±5.38	109.38±4.21
		peak 2	168.89±0.12	172.55±0.09	2.42±0.34
		peak 3	248.15±0.46	250.49±0.48	97.51±2.46
		peak 4*	253.86±0.26	256.08±0.20	19.83±1.25
14	Heviran 800	peak 1	94.12±1.31	118.71±1.95	111.06±5.72
		peak 2	169.95±0.61	173.45±0.16	1.08±0.12
		peak 3	246.51±0.52	250.23±0.18	100.75±1.67
		peak 4*	253.31±0.13	255.54±0.09	15.70±1.17
15	Nifuroksazyd Hasco	peak 1*	267.72±0.78	278.77±0.62	301.26±10.12
16	Nifuroksazyd 200 Hasco	peak 1*	268.55±0.70	278.04±0.65	318.08±18.05
17	Nifuroksazyd Richter	peak 1*	270.94±0.93	277.97±0.16	287.80±18.08
18	Paracetamol Polfa-Łódź	peak 1	92.99±0.30	97.95±0.20	35.99±5.46
		peak 2	151.80±0.27	159.41±0.90	107.66±3.74
19	Furosemidum Polpharma	peak 1	140.30±0.73	147.45±0.22	25.36±1.01
		peak 2	197.91±0.80	208.90±0.27	77.72±1.29
		peak 3*	214.78±0.11	218.91±0.26	46.54±2.49
20	Furosemidum Polfarmex	peak 1	144.10±0.22	148.76±0.28	31.89±1.05
		peak 2	195.53±1.43	206.80±0.24	72.87±8.77
		peak 3*	212.11±0.34	214.64±0.62	30.42±0.94
21	Encorton 5 mg	peak 1	143.43±1.21	148.71±0.31	108.21±5.44
		peak 2	203.60±0.81	211.72±0.23	91.41±7.75
22	Encorton 10 mg	peak 1	144.40±1.12	148.86±0.73	99.90±15.87
		peak 2	204.51±0.30	212.08±0.22	90.33±15.31
23	Encorton 20 mg	peak 1	144.94±0.62	148.37±0.64	104.15±5.65
		peak 2	204.15±0.28	211.96±0.45	93.81±8.42
24	Spironol 25	peak 1	143.96±1.32	148.10±0.45	82.09±10.18
		peak 2	200.44±0.15	210.41±0.38	70.87±1.74
25	Spironol 100	peak 1	142.27±0.12	147.41±0.04	73.60±1.88
		peak 2	198.42±0.06	209.21±0.04	66.62±1.66
26	Metoclopramidum	peak 1	132.16±0.22	144.24±0.88	48.98±4.33
		peak 2	187.93±1.18	206.10±0.44	143.24±5.18
27	Enarenal 5	peak 1	135.05±1.76	145.91±0.32	110.74±14.25
		peak 2	169.45±5.96	189.47±0.75	143.38±18.71
28	Enarenal 10	peak 1	136.32±0.62	144.76±1.36	92.99±1.30
		peak 2	170.52±5.75	191.12±2.21	143.53±21.11
29	Enarenal 20	peak 1	138.07±0.03	147.07±0.45	104.91±2.38
		peak 2	173.40±1.03	189.19±0.32	127.00±7.49
30	Etopiryna	peak 1	72.01±0.47	78.70±0.53	30.53±3.06
31	Tialorid	peak 1	141.12±0.39	143.92±0.86	121.77±0.64
		peak 2	201.56±0.65	207.63±0.05	179.61±1.35
32	Tialorid mite	peak 1	135.96±0.74	144.72±0.51	51.06±5.93

		peak 2	199.00±0.84	208.12±0.22	79.78±4.81
33	Luminalum Unia 15	peak 1	141.45±0.30	144.11±0.33	100.96±6.28
		peak 2	169.14±0.20	171.70±0.25	14.52±1.70
		peak 3	200.03±0.34	207.41±0.35	88.87±9.34
34	Luminalum Unia 100	peak 1	141.33±0.16	145.87±0.43	47.20±3.41
		peak 2	169.98±0.25	173.22±0.23	69.24±6.28
		peak 3	192.19±0.41	202.71±0.07	77.56±4.99

*exothermic peak

Table S2. Results of the DSC measurements for all drug products obtained during the second series of experiments (March 2012).

No	Trade names of drug products	Peak number	Temperature and enthalpy values for phase transitions (mean ± standard deviations, n = 3)		
			March 2012		
			T_{on} (°C)	T_p (°C)	ΔH_f (J/g)
1	Theospirex retard 150	peak 1	267.72±0.25	269.57±0.31	127.83±2.42
2	Theospirex retard 300	peak 1	267.74±0.19	270.16±0.28	125.70±4.04
3	Paracetamol Biofarm	peak 1	167.57±0.22	170.57±0.38	151.47±4.25
4	Paracetamol Aflofarm	peak 1	167.61±0.39	171.18±1.02	144.50±2.56
5	Pyralgina	peak 1	101.15±2.41	125.27±6.11	141.92±6.54
		peak 2	216.97±0.25	223.00±1.74	45.55±2.02
		peak 3*	225.19±0.47	231.72±0.24	287.81±1.95
6	Ranigast Polpharma	peak 1	144.19±0.54	147.57±0.24	56.50±2.14
		peak 2*	150.20±0.39	154.19±0.51	290.91±6.52
7	Ranigast Max	peak 1	143.56±0.55	147.23±0.19	61.08±2.11
		peak 2*	149.65±0.61	153.51±0.95	293.16±5.45
8	Cyclonamine	peak 1	127.75±0.23	129.89±0.40	118.70±1.99
9	Cipronex 250	peak 1	122.37±0.54	144.84±1.06	82.77±3.45
		peak 2	280.16±4.45	287.89±4.11	10.25±2.08
10	Cipronex 500	peak 1	119.92±1.52	145.52±0.47	92.72±9.74
		peak 2	282.22±1.83	288.28±1.02	10.54±1.47
11	Coffepirine	peak 1	104.74±10.14	126.74±1.79	137.30±4.07
12	Heviran 200	peak 1	118.53±2.54	144.81±0.54	116.10±3.14
		peak 2	170.28±1.47	173.55±0.22	5.64±1.15
		peak 3	246.03±6.15	249.23±0.87	95.74±0.45
		peak 4*	252.26±4.78	254.49±0.33	17.97±0.79
13	Heviran 400	peak 1	101.17±0.14	128.36±0.84	110.79±1.65
		peak 2	169.14±0.36	172.97±0.12	2.23±1.15
		peak 3	247.93±0.38	251.36±0.61	95.30±2.45
		peak 4*	255.32±0.33	257.39±0.31	17.70±1.43
14	Heviran 800	peak 1	95.84±0.28	121.88±0.27	106.25±2.57
		peak 2	170.41±0.52	174.01±0.22	1.59±0.96

		peak 3	247.59±0.32	251.15±0.96	99.51±1.83
		peak 4*	255.17±0.25	257.26±0.41	15.71±1.45
15	Nifuroksazyd Hasco	peak 1*	267.35±0.76	279.04±0.34	259.37±4.54
16	Nifuroksazyd 200 Hasco	peak 1*	268.96±0.54	276.87±0.41	328.72±14.01
17	Nifuroksazyd Richter	peak 1*	270.55±2.62	278.52±3.80	234.00±16.54
18	Paracetamol Polfa-Łódź	peak 1	93.63±0.21	98.80±0.09	38.22±1.88
		peak 2	151.60±0.74	159.98±0.13	97.41±2.46
19	Furosemidum Polpharma	peak 1	141.95±0.96	147.83±0.36	26.34±2.48
		peak 2	200.04±0.68	209.35±0.65	80.48±1.33
		peak 3*	215.67±0.08	219.68±0.39	43.21±3.15
20	Furosemidum Polfarmex	peak 1	144.27±0.21	150.17±0.33	28.72±2.26
		peak 2	196.99±0.85	207.40±0.66	78.42±3.47
		peak 3*	213.11±1.58	215.63±0.48	28.96±1.94
21	Encorton 5 mg	peak 1	143.13±0.18	146.59±0.55	106.28±1.07
		peak 2	203.45±0.26	211.24±0.20	92.66±1.87
22	Encorton 10 mg	peak 1	144.83±0.36	148.83±0.54	96.63±6.18
		peak 2	204.83±0.45	211.81±0.39	88.32±9.01
23	Encorton 20 mg	peak 1	142.82±0.48	149.12±0.47	99.77±4.95
		peak 2	203.63±0.61	211.19±0.24	80.32±9.24
24	Spironol 25	peak 1	143.93±0.59	148.01±0.78	79.65±5.46
		peak 2	201.02±0.91	211.36±0.13	69.30±2.86
25	Spironol 100	peak 1	142.77±0.20	147.91±0.60	70.14±2.32
		peak 2	198.88±0.94	209.72±0.44	65.85±3.07
26	Metoclopramidum	peak 1	136.02±0.56	145.85±0.67	45.82±2.98
		peak 2	186.95±0.84	205.45±0.82	131.05±6.87
27	Enarenal 5	peak 1	134.57±1.68	147.76±0.41	109.27±11.19
		peak 2	168.55±3.12	192.41±0.81	142.02±11.76
28	Enarenal 10	peak 1	136.59±0.94	146.27±1.16	92.18±4.95
		peak 2	169.02±2.27	189.01±1.41	145.50±16.02
29	Enarenal 20	peak 1	139.43±1.24	146.60±2.03	101.77±4.40
		peak 2	170.97±0.62	186.16±4.01	134.15±7.46
30	Etopiryna	peak 1	71.18±0.51	78.18±0.77	31.91±3.16
31	Tialorid	peak 1	141.27±0.64	143.89±0.87	125.26±2.19
		peak 2	201.93±0.55	207.54±0.58	185.81±2.72
32	Tialorid mite	peak 1	137.91±0.80	145.82±0.44	51.81±1.89
		peak 2	199.21±1.01	208.85±1.74	78.22±2.41
33	Luminalum Unia 15	peak 1	141.97±0.41	144.81±0.32	101.45±1.13
		peak 2	169.54±0.28	172.20±0.42	14.88±0.98
		peak 3	199.48±0.26	207.31±0.34	86.95±6.09
34	Luminalum Unia 100	peak 1	141.68±0.19	145.94±0.38	43.26±4.15
		peak 2	170.11±0.36	173.22±0.29	58.69±6.06
		peak 3	192.23±0.44	203.14±0.51	73.32±5.21

*exothermic peak

Table S3. Results of the DSC measurements for all drug products obtained during the third series of experiments (September 2013).

No	Trade names of drug products	Peak number	Temperature and enthalpy values for phase transitions (mean ± standard deviations, n = 3)		
			September 2013		
			T_{on} (°C)	T_p (°C)	ΔH_f (J/g)
1	Theospirex retard 150	peak 1	268.05±0.33	270.43±0.26	112.21±1.98
2	Theospirex retard 300	peak 1	266.26±0.12	269.48±0.47	124.15±3.21
3	Paracetamol Biofarm	peak 1	167.54±0.19	170.11±0.27	146.82±5.14
4	Paracetamol Aflofarm	peak 1	167.59±0.17	170.82±0.38	149.35±4.14
5	Pyralgina	peak 1	103.21±0.98	129.09±3.28	138.59±2.78
		peak 2	217.16±1.24	222.98±2.15	44.55±0.95
		peak 3*	225.01±0.09	231.33±0.32	278.93±2.67
6	Ranigast Polpharma	peak 1	141.83±0.41	146.05±0.34	54.14±3.25
		peak 2*	148.37±0.96	153.27±0.57	297.56±7.54
7	Ranigast Max	peak 1	143.62±0.31	147.35±0.36	59.95±4.04
		peak 2*	149.76±0.62	153.76±0.41	291.79±4.46
8	Cyclonamine	peak 1	127.85±0.15	129.41±0.19	108.59±2.10
9	Cipronex 250	peak 1	113.60±0.87	146.87±0.64	82.14±6.45
		peak 2	280.2±0.39	286.9±1.97	9.24±3.15
10	Cipronex 500	peak 1	126.12±0.21	146.36±0.97	91.21±5.96
		peak 2	281.16±0.24	286.41±1.08	11.14±3.22
11	Coffepirine	peak 1	112.22±7.72	125.88±2.45	141.99±6.17
12	Heviran 200	peak 1	115.93±0.11	144.52±2.15	119.24±4.78
		peak 2	170.87±0.98	174.27±0.96	4.88±1.02
		peak 3	246.03±0.25	249.23±0.34	95.74±1.04
		peak 4*	252.26±1.69	254.49±0.88	17.94±1.15
13	Heviran 400	peak 1	94.92±1.27	122.46±0.68	109.77±2.26
		peak 2	169.26±0.09	172.87±0.73	1.99±0.94
		peak 3	249.23±0.71	251.59±0.17	93.48±1.94
		peak 4*	255.27±0.14	257.41±0.37	18.41±2.22
14	Heviran 800	peak 1	100.83±0.19	127.79±0.28	116.45±2.25
		peak 2	169.89±0.45	173.55±0.11	1.89±0.54
		peak 3	247.44±0.34	250.26±0.08	96.72±1.21
		peak 4*	253.49±0.47	255.43±0.85	18.38±2.06
15	Nifuroksazyd Hasco	peak 1*	268.11±0.75	277.24±0.64	289.05±8.43
16	Nifuroksazyd 200 Hasco	peak 1*	268.65±1.45	278.20±0.19	275.62±3.69
17	Nifuroksazyd Richter	peak 1*	270.84±2.62	278.58±3.16	253.14±15.57
18	Paracetamol Polfa-Łódź	peak 1	93.87±0.57	98.26±1.01	37.92±1.15
		peak 2	151.85±0.62	159.44±0.99	98.22±1.64

19	Furosemidum Polpharma	peak 1	141.52±0.12	148.55±0.38	27.64±2.49
		peak 2	198.42±0.16	209.58±0.37	70.98±2.35
		peak 3*	215.04±1.17	219.81±0.87	44.10±1.88
20	Furosemidum Polfarmex	peak 1	145.11±0.64	150.18±0.47	26.40±2.74
		peak 2	192.57±0.87	208.08±2.04	71.02±4.05
		peak 3*	213.66±0.20	216.92±0.90	29.46±2.08
21	Encorton 5 mg	peak 1	143.39±0.74	149.17±0.68	105.67±4.12
		peak 2	205.19±2.21	211.98±1.34	82.63±6.97
22	Encorton 10 mg	peak 1	144.53±0.87	150.29±0.90	89.65±8.94
		peak 2	205.24±0.52	212.53±0.87	76.75±15.87
23	Encorton 20 mg	peak 1	145.16±0.30	149.53±1.04	99.82±10.01
		peak 2	204.84±0.43	212.15±0.94	77.84±7.87
24	Spironol 25	peak 1	144.24±0.22	149.02±0.74	78.62±11.12
		peak 2	199.17±0.47	209.82±0.62	69.28±2.54
25	Spironol 100	peak 1	142.57±0.38	147.90±0.66	70.99±0.77
		peak 2	197.89±0.33	208.67±0.72	65.82±1.14
26	Metoclopramidum	peak 1	139.51±0.66	146.97±0.28	45.64±1.94
		peak 2	188.2±1.05	222.9±0.87	129.8±6.97
27	Enarenal 5	peak 1	138.79±1.54	147.98±0.77	111.64±3.18
		peak 2	168.55±0.96	174.40±0.68	138.37±16.51
28	Enarenal 10	peak 1	137.47±0.11	147.01±2.02	91.94±9.44
		peak 2	168.04±3.34	192.14±1.66	148.76±5.53
29	Enarenal 20	peak 1	139.82±0.91	148.35±0.80	104.85±6.91
		peak 2	169.87±0.45	192.02±1.11	138.41±6.17
30	Etopiryna	peak 1	68.00±0.66	76.33±0.14	32.92±4.04
31	Tialorid	peak 1	142.36±0.33	146.23±0.37	123.72±2.47
		peak 2	201.62±0.61	208.10±0.76	169.24±3.06
32	Tialorid mite	peak 1	139.94±0.07	146.79±1.20	50.94±2.31
		peak 2	197.68±0.23	209.00±0.82	72.82±3.05
33	Luminalum Unia 15	peak 1	141.76±0.60	146.56±0.17	101.11±5.29
		peak 2	169.23±0.23	171.80±0.41	13.92±2.04
		peak 3	200.77±1.04	207.44±0.88	83.27±6.22
34	Luminalum Unia 100	peak 1	141.83±0.52	145.74±0.11	42.67±2.98
		peak 2	170.10±0.28	172.93±0.24	59.71±4.66
		peak 3	191.39±0.33	201.78±0.32	74.43±5.11

*exothermic peak

Table S4. Results of the DSC measurements for all drug products obtained during the fourth series of experiments (December 2014).

No	Trade names of drug products	Peak number	Temperature and enthalpy values for phase transitions (mean ± standard deviations, n = 3)		
			December 2014		
			T_{on} (°C)	T_p (°C)	ΔH_f (J/g)
1	Theospirex retard 150	peak 1	268.05±0.29	271.43±0.36	118.03±2.98
2	Theospirex retard 300	peak 1	267.70±0.36	270.60±0.51	126.70±4.12
3	Paracetamol Biofarm	peak 1	167.49±0.14	170.86±0.22	153.40±2.47
4	Paracetamol Aflofarm	peak 1	167.51±0.13	171.21±0.55	149.51±6.15
5	Pyralgina	peak 1	103.50±6.02	126.78±4.96	139.17±2.08
		peak 2	211.14±3.87	222.06±1.89	39.90±1.24
		peak 3*	233.97±0.61	231.07±0.44	281.24±4.54
6	Ranigast Polpharma	peak 1	141.48±0.52	145.10±0.80	52.67±2.51
		peak 2*	148.37±1.91	153.27±0.38	296.68±4.57
7	Ranigast Max	peak 1	143.48±1.04	147.22±0.47	60.19±2.77
		peak 2*	149.45±0.09	153.31±1.03	292.52±6.18
8	Cyclonamine	peak 1	127.51±0.11	129.65±0.20	111.98±1.41
9	Cipronex 250	peak 1	124.01±5.87	145.59±2.54	85.35±3.36
		peak 2	279.54±4.96	285.44±3.22	10.98±2.44
10	Cipronex 500	peak 1	122.15±3.25	145.69±0.98	94.49±4.79
		peak 2	278.97±2.78	287.54±1.74	9.85±3.01
11	Coffepirine	peak 1	111.22±11.48	124.41±3.55	141.82±4.82
12	Heviran 200	peak 1	115.93±4.54	144.52±5.44	119.24±4.85
		peak 2	170.87±1.97	174.27±0.87	4.88±1.05
		peak 3	240.01±1.08	247.28±0.31	92.62±1.08
		peak 4*	250.41±0.44	252.55±0.19	14.4±1.11
13	Heviran 400	peak 1	108.69±2.65	134.82±0.87	109.77±2.93
		peak 2	170.04±0.62	173.44±0.24	1.28±0.42
		peak 3	243.81±1.06	247.74±0.49	97.03±3.69
		peak 4*	251.22±2.16	253.52±0.54	18.22±1.18
14	Heviran 800	peak 1	92.57±0.69	119.36±1.21	110.78±0.74
		peak 2	170.93±0.54	174.14±0.59	1.80±0.98
		peak 3	244.75±0.57	248.74±0.34	98.05±0.66
		peak 4*	251.86±2.42	254.21±3.11	15.1±1.64
15	Nifuroksazyd Hasco	peak 1*	271.64±1.47	281.30±1.16	262.37±24.81
16	Nifuroksazyd 200 Hasco	peak 1*	272.14±0.61	279.85±0.82	329.71±38.15
17	Nifuroksazyd Richter	peak 1*	271.43±2.34	278.26±1.07	244.42±19.12
18	Paracetamol Polfa-Łódź	peak 1	92.59±0.21	98.13±0.13	35.58±2.57
		peak 2	152.25±0.55	159.31±0.47	100.08±2.91
19	Furosemidum Polpharma	peak 1	142.93±0.98	148.62±0.44	27.80±1.51

		peak 2	200.24±1.23	209.97±0.89	81.61±3.03
		peak 3*	216.14±0.25	219.79±0.46	39.40±1.31
20	Furosemidum Polfarmex	peak 1	144.71±1.16	150.14±0.92	34.92±2.11
		peak 2	196.64±1.30	208.02±1.54	85.09±4.61
		peak 3*	213.39±2.29	216.10±2.02	32.40±1.20
21	Encorton 5 mg	peak 1	143.59±1.04	150.13±0.57	110.94±6.16
		peak 2	204.02±0.95	212.02±0.72	85.28±4.02
22	Encorton 10 mg	peak 1	145.07±1.49	149.84±0.51	97.51±4.15
		peak 2	205.21±0.81	213.31±0.90	87.32±1.72
23	Encorton 20 mg	peak 1	145.87±0.67	149.37±0.37	100.31±3.18
		peak 2	204.60±0.39	212.89±0.42	81.52±1.01
24	Spironol 25	peak 1	144.77±0.29	148.57±0.63	87.72±3.84
		peak 2	201.68±0.36	212.16±0.18	66.36±1.24
25	Spironol 100	peak 1	143.33±0.60	148.05±0.43	68.05±2.83
		peak 2	199.72±0.67	210.88±0.81	63.46±3.11
26	Metoclopramidum	peak 1	139.02±1.04	146.14±0.14	46.03±1.18
		peak 2	187.45±0.85	217.22±0.07	137.98±2.12
27	Enarenal 5	peak 1	138.93±0.90	147.99±0.67	118.91±4.55
		peak 2	168.65±1.31	190.44±0.87	134.08±16.11
28	Enarenal 10	peak 1	137.08±0.12	147.03±0.33	94.88±2.49
		peak 2	167.49±1.02	189.18±1.24	142.18±14.19
29	Enarenal 20	peak 1	137.21±1.10	148.60±0.82	105.44±8.47
		peak 2	166.05±3.04	191.16±2.47	140.62±9.74
30	Etopiryna	peak 1	70.58±0.90	78.20±0.54	30.35±1.41
31	Tialorid	peak 1	141.01±0.21	143.72±0.30	125.55±2.18
		peak 2	201.68±1.14	207.88±1.05	172.65±16.22
32	Tialorid mite	peak 1	142.28±0.69	146.91±0.40	51.20±3.42
		peak 2	199.58±1.03	208.66±0.91	73.38±2.92
33	Luminalum Unia 15	peak 1	142.81±0.23	145.04±0.33	104.97±3.47
		peak 2	169.44±0.26	172.20±0.64	14.00±0.81
		peak 3	200.26±0.05	207.38±0.49	82.44±2.04
34	Luminalum Unia 100	peak 1	142.26±0.28	145.38±0.61	43.04±1.48
		peak 2	170.51±0.34	173.28±0.45	60.58±4.82
		peak 3	192.93±0.52	204.30±0.76	79.79±2.12

*exothermic peak

Table S5. Results of the DSC measurements for all drug products obtained during the fifth series of experiments (November 2016).

No	Trade names of drug products	Peak number	Temperature and enthalpy values for phase transitions (mean ± standard deviations, n = 3)		
			November 2016		
			T_{on} (°C)	T_p (°C)	ΔH_f (J/g)
1	Theospirex retard 150	peak 1	269.34 ±0.51	272.10±0.48	114.66±3.32
2	Theospirex retard 300	peak 1	267.77±1.81	271.43±1.41	124.79±3.67
3	Paracetamol Biofarm	peak 1	168.84±0.29	172.07±0.58	150.41±3.47
4	Paracetamol Aflofarm	peak 1	168.62±0.07	172.18±1.19	145.69±9.05
5	Pyralgina	peak 1	109.65±4.54	133.90±3.68	137.84±2.43
		peak 2	219.24±0.54	225.14±0.42	39.87±2.24
		peak 3*	227.25±0.45	234.10±0.30	281.13±6.67
6	Ranigast Polpharma	peak 1	143.31±1.67	147.45±1.14	53.29±4.07
		peak 2*	149.91±1.32	154.24±0.77	293.30±1.32
7	Ranigast Max	peak 1	144.36±0.30	148.41±0.17	59.99±3.32
		peak 2*	151.14±0.20	154.76±0.11	294.74±3.47
8	Cyclonamine	peak 1	128.78±0.08	131.21±0.16	105.45±1.61
9	Cipronex 250	peak 1	123.44±0.63	146.82±0.34	85.21±1.53
		peak 2	282.21±1.17	288.11±0.74	10.01±2.54
10	Cipronex 500	peak 1	126.35±2.10	148.03±0.17	93.68±1.37
		peak 2	280.30±0.56	286.32±0.84	11.72±2.03
11	Coffepirine	peak 1	115.29±14.58	127.81±1.70	132.89±4.24
12	Heviran 200	peak 1	100.32±5.09	125.90±10.28	119.10±3.41
		peak 2	171.08±0.34	175.33±0.29	4.14±0.38
		peak 3	247.86±0.46	252.06±0.11	95.30±0.52
		peak 4*	255.26±0.12	257.41±0.04	16.14±0.79
13	Heviran 400	peak 1	102.57±2.29	130.65±2.95	109.48±0.54
		peak 2	171.26±0.21	174.68±0.61	2.14±0.46
		peak 3	250.23±0.07	253.11±0.11	92.57±1.36
		peak 4*	256.78±0.15	259.05±0.06	16.84±0.28
14	Heviran 800	peak 1	106.88±0.69	132.96±0.45	110.91±1.06
		peak 2	171.46±0.11	175.06±0.39	1.77±0.64
		peak 3	247.83±0.38	251.54±0.19	100.40±0.84
		peak 4*	254.76±2.69	257.10±1.93	17.54±1.05
15	Nifuroksazyd Hasco	peak 1*	271.16±1.12	281.51±1.19	302.24±26.83
16	Nifuroksazyd 200 Hasco	peak 1*	270.86±0.68	281.12±0.58	305.12±47.01
17	Nifuroksazyd Richter	peak 1*	273.58±2.13	280.49±0.99	280.36±18.90
18	Paracetamol Polfa-Łódź	peak 1	94.57±0.20	99.55±0.25	34.50±3.62
		peak 2	152.94±0.45	160.62±0.60	101.19±3.32
19	Furosemidum Polpharma	peak 1	145.69±1.33	149.92±0.23	27.56±1.43

		peak 2	200.98±2.11	211.40±0.15	83.64±3.48
		peak 3*	217.89±0.07	221.63±0.36	41.38±1.24
20	Furosemidum Polfarmex	peak 1	145.89±1.76	150.44±1.02	34.18±3.21
		peak 2	200.55±2.23	211.10±0.65	80.28±5.65
		peak 3*	216.30±2.94	219.73±3.29	34.07±1.16
21	Encorton 5 mg	peak 1	147.45±0.36	150.78±0.42	96.25±1.23
		peak 2	206.54±0.12	214.10±0.14	88.80±4.18
22	Encorton 10 mg	peak 1	147.32±1.89	150.81±0.36	92.93±7.09
		peak 2	206.03±2.28	214.48±1.40	81.62±5.88
23	Encorton 20 mg	peak 1	145.37±0.65	151.68±0.06	91.14±1.67
		peak 2	205.59±0.54	214.06±0.29	77.20±2.80
24	Spironol 25	peak 1	146.17±0.25	149.95±0.16	77.96±3.55
		peak 2	203.36±0.41	213.66±0.03	60.92±0.91
25	Spironol 100	peak 1	144.35±0.56	149.17±0.46	66.90±4.96
		peak 2	200.79±0.32	211.89±0.73	63.10±2.73
26	Metoclopramidum	peak 1	140.54±0.28	146.93±1.02	47.08±0.66
		peak 2	189.03±0.55	215.12±0.90	136.94±3.88
27	Enarenal 5	peak 1	138.50±2.77	147.78±0.25	105.01±21.77
		peak 2	165.84±2.69	189.01±0.70	144.00±10.41
28	Enarenal 10	peak 1	138.52±0.16	147.37±0.48	92.18±1.71
		peak 2	168.95±2.95	192.05±4.46	140.63±11.34
29	Enarenal 20	peak 1	139.89±3.14	149.02±0.36	108.85±10.41
		peak 2	168.14±0.95	189.12±1.44	138.71±7.62
30	Etopiryna	peak 1	71.21±1.94	78.86±1.00	27.78±1.87
31	Tialorid	peak 1	143.04±0.09	146.02±0.20	125.90±1.97
		peak 2	203.69±1.09	209.76±0.17	168.17±5.32
32	Tialorid mite	peak 1	144.79±0.26	148.57±0.33	49.95±3.13
		peak 2	201.74±0.20	210.42±0.28	73.55±3.56
33	Luminalum Unia 15	peak 1	143.36±0.16	146.10±0.34	99.89±3.24
		peak 2	170.49±0.17	173.46±0.18	14.44±0.45
		peak 3	201.47±0.25	208.90±0.10	85.83±2.52
34	Luminalum Unia 100	peak 1	143.19±0.32	147.66±0.50	42.08±1.98
		peak 2	171.87±0.31	175.14±0.47	57.93±1.24
		peak 3	194.60±0.79	205.18±0.53	72.88±1.60

*exothermic peak

Table S6. Results of the DSC measurements for all drug products obtained during the sixth series of experiments (July 2022).

No	Trade names of drug products	Peak number	Temperature and enthalpy values for phase transitions (mean ± standard deviations, n = 3)		
			July 2022		
			T_{on} (°C)	T_p (°C)	ΔH_f (J/g)
1	Theospirex retard 150	peak 1	269.23±0.15	271.65±0.30	119.01±2.23
2	Theospirex retard 300	peak 1	269.89±0.47	272.37±0.41	126.01±5.18
3	Paracetamol Biofarm	peak 1	168.33±0.18	171.88±0.61	153.72±4.25
4	Paracetamol Aflofarm	peak 1	168.48±0.38	170.68±0.54	149.30±2.93
5	Pyralgina	peak 1	119.71±3.91	141.01±3.15	136.76±0.67
		peak 2	218.69±0.14	224.21±0.02	39.61±1.94
		peak 3*	226.36±0.13	232.53±0.59	280.73±3.85
6	Ranigast Polpharma	peak 1	144.09±0.14	147.86±0.28	56.30±0.85
		peak 2*	150.35±0.39	154.13±0.18	298.92±3.83
7	Ranigast Max	peak 1	143.41±0.75	147.56±0.53	61.16±7.63
		peak 2*	150.13±0.52	154.16±0.21	296.55±4.30
8	Cyclonamine	peak 1	128.31±0.09	130.50±0.12	110.28±2.74
9	Cipronex 250	peak 1	130.88±9.91	145.56±0.10	85.64±1.04
		peak 2	281.59±2.15	287.52±1.07	11.71±1.96
10	Cipronex 500	peak 1	126.60±5.10	146.29±0.73	93.87±0.72
		peak 2	281.16±0.63	286.18±0.94	11.90±1.78
11	Coffepirine	peak 1	116.38±14.13	128.13±1.78	142.38±1.85
12	Heviran 200	peak 1	101.72±3.07	130.48±3.90	116.83±1.53
		peak 2	170.34±0.18	174.28±0.18	4.24±0.63
		peak 3	247.16±0.79	251.26±0.13	97.51±0.35
		peak 4*	254.12±0.24	256.33±0.33	16.25±1.39
13	Heviran 400	peak 1	99.74±7.25	123.10±5.02	110.44±0.77
		peak 2	169.74±0.14	173.64±0.21	2.05±0.09
		peak 3	249.40±0.67	252.09±0.41	95.22±0.86
		peak 4*	255.55±0.54	257.68±0.57	17.29±0.82
14	Heviran 800	peak 1	101.12±0.71	124.41±0.68	108.65±0.92
		peak 2	171.25±0.54	174.59±0.46	1.57±0.47
		peak 3	247.88±0.44	251.73±0.41	100.25±1.23
		peak 4*	254.91±2.14	257.05±1.61	17.65±1.15
15	Nifuroksazyd Hasco	peak 1*	270.70±1.44	280.89±0.53	320.38±4.61
16	Nifuroksazyd 200 Hasco	peak 1*	270.00±0.43	280.85±0.58	326.86±43.21
17	Nifuroksazyd Richter	peak 1*	271.34±1.30	278.32±0.54	303.10±18.15
18	Paracetamol Polfa-Łódź	peak 1	92.44±2.16	97.73±1.24	35.00±2.28
		peak 2	152.56±0.14	159.62±0.30	101.83±1.66
19	Furosemidum Polpharma	peak 1	143.96±0.42	149.26±0.21	28.14±1.16

		peak 2	201.80±0.70	211.56±0.30	85.65±1.77
		peak 3*	217.96±0.31	221.69±0.37	42.82±1.35
20	Furosemidum Polfarmex	peak 1	146.37±1.26	151.20±0.47	32.82±2.07
		peak 2	197.64±0.58	207.88±1.24	80.32±4.20
		peak 3*	213.90±1.03	216.48±1.17	26.43±1.29
21	Encorton 5 mg	peak 1	146.28±1.36	149.40±0.70	99.19±5.95
		peak 2	205.40±1.27	213.38±0.80	94.36±3.83
22	Encorton 10 mg	peak 1	146.38±0.84	149.36±0.46	98.50±1.45
		peak 2	205.83±0.19	213.41±0.06	94.76±2.96
23	Encorton 20 mg	peak 1	146.71±0.64	149.50±0.55	101.02±4.89
		peak 2	205.70±0.20	213.27±0.12	90.03±1.72
24	Spironol 25	peak 1	145.36±0.36	149.15±0.34	84.59±2.35
		peak 2	203.06±0.38	213.24±0.24	67.34±1.52
25	Spironol 100	peak 1	143.71±0.28	148.72±0.36	67.61±0.95
		peak 2	200.63±0.80	211.81±0.84	63.24±3.90
26	Metoclopramidum	peak 1	139.25±2.19	146.54±1.24	47.35±0.92
		peak 2	188.31±0.71	216.19±0.16	139.66±2.47
27	Enarenal 5	peak 1	138.05±0.47	147.18±0.30	110.70±8.03
		peak 2	167.73±0.88	170.60±0.54	135.49±12.72
28	Enarenal 10	peak 1	138.68±0.40	147.11±0.20	92.87±1.00
		peak 2	167.94±5.87	171.75±1.71	136.24±10.97
29	Enarenal 20	peak 1	140.18±2.05	148.76±0.29	103.39±11.04
		peak 2	167.36±1.40	170.22±0.98	138.92±5.13
30	Etopiryna	peak 1	71.07±0.32	78.39±0.10	27.07±1.35
31	Tialorid	peak 1	141.82±0.15	144.61±0.29	124.20±2.87
		peak 2	202.77±0.48	209.29±0.47	171.60±18.00
32	Tialorid mite	peak 1	143.37±0.96	148.09±0.17	50.91±3.69
		peak 2	200.98±1.43	209.79±0.09	75.03±1.63
33	Luminalum Unia 15	peak 1	143.27±0.04	145.74±0.27	100.69±0.62
		peak 2	170.25±0.07	173.50±0.87	15.04±0.09
		peak 3	200.63±0.20	208.41±0.17	83.90±2.89
34	Luminalum Unia 100	peak 1	142.47±0.35	146.55±0.56	43.52±1.71
		peak 2	171.05±0.10	174.06±0.09	59.02±4.52
		peak 3	193.71±0.26	204.35±0.08	71.66±1.49

*exothermic peak
